# SPAIRE Approach Versus Direct Anterior Approach in Hemiarthroplasty for Displaced Femoral Neck Fractures: A Randomized Controlled Trial

**DOI:** 10.2106/JBJS.OA.26.00171

**Published:** 2026-07-13

**Authors:** Stein H. Ugland, Oystein T. Fagerberg, Terje O. Ugland, Knut E. Mjaaland, Are H. Pripp, Lars Nordsletten

**Affiliations:** 1 Department of Orthopaedics, Sorlandet Hospital, Kristiansand, Norway; 2 Institute of Clinical Medicine, University of Oslo, Oslo, Norway; 3 Department of Orthopaedics, Sorlandet Hospital, Arendal, Norway; 4 Oslo Centre of Biostatistics and Epidemiology, Oslo, Norway; 5 Division of Orthopaedic Surgery, Oslo University Hospital, Oslo, Norway

## Abstract

**Background:**

Several surgical approaches are used for hemiarthroplasty (HA) in femoral neck fractures (FNF). The Sparing Piriformis and Obturator Internus, Repair Externus (SPAIRE) approach, introduced in the Norwegian Hip Fracture Register in 2019, is gaining popularity, accounting for 18% of HAs in Norway in 2024. However, supporting evidence remains limited. This randomized controlled trial (RCT) aimed at comparing the SPAIRE and direct anterior (DA) approaches in patients with FNF treated with HA.

**Methods:**

158 patients with a displaced FNF operated with HA were from January 2022 to June 2024 included in this RCT at Sorlandet Hospital Kristiansand and Sorlandet Hospital Arendal and randomized to the SPAIRE (n = 79) or the DA approach (n = 79) and followed for 12 months. The mean age was 79 years (70-90), and 59% were women. Physiotherapists blinded to allocation collected functional outcomes and patient-reported outcome measures postoperatively and at 3 and 12 months.

**Results:**

158 patients were included and 126 were assessed at final follow-up. All patients received the allocated treatment, and the randomization groups were similar. The duration of surgery was 52 min in the SPAIRE group vs. 63 minutes in the DA group (mean difference 9 minutes, 95% confidence interval [CI] 8-15; p < 0.01). At 12 months, the mean Harris Hip Score was 86 and 88, respectively (mean difference 2.3, 95% CI −2.7 to 7.3; p = 0.365). We did not find clinically relevant differences in patient-reported outcome measures between the groups at any time point. Nine complications requiring reoperation were recorded in 8 patients.

**Conclusion:**

No significant differences in clinical outcomes were found between patients operated with the DA or SPAIRE approach. SPAIRE approach may be an alternative for posterior approach surgeons performing HA on FNF patients. However, larger studies are warranted to establish the clinical implications of the approach.

**Level of Evidence:**

Level II. See Instructions for Authors for a complete description of levels of evidence.

## Background

Nearly 9,000 proximal femur fractures occur annually in Norway, and approximately 4,600 of these are femoral neck fractures (FNF)^1^. National Norwegian guidelines recommend that displaced medial FNFs are operated with arthroplasty, preferably hemiarthroplasty (HA). 4100 HA’s were implanted due to FNF in 2024^1^. Owing to advantages and disadvantages regarding patient-reported outcome measures (PROM) and complications, no consensus exists regarding the optimal approach. The posterior approach (PA) is widely used in total hip arthroplasty (THA) but is associated with an increased dislocation risk in HA^2,3^. This has resulted in Norwegian guidelines recommending against using the PA in HA. The direct lateral (DL) approach has dominated in HA due to a low dislocation risk but is increasingly unpopular in THA^4^. This is mainly due to an increased risk of trochanteric pain and limping due to damaged hip abductors^5-7^. The direct anterior (DA) and the modified anterolateral approaches have gained popularity due to good clinical results and its muscle-sparing concept^8,9^.

The SPAIRE approach appeared in the Norwegian Hip Fracture register in 2019^1^. It is becoming increasingly popular due to indications of reduced dislocation risk in HA^10-12^. A few reports on the SPAIRE approach have been published. Charity et al. reported 1 dislocation with the SPAIRE approach in a series of 285 HA’s over a 3.5-year period^13^. Nakamura et al. did not report dislocations in a multicenter study of 322 patients with FNF operated with HA^10^. Ball et al., in their randomized controlled trial (RCT), compared the DL and the SPAIRE approach in patients with FNF operated with HA and reported 1 dislocation in each group^11^. These findings indicate that orthopaedic surgeons routinely using the PA in THA may consider the SPAIRE approach for HA in patients with FNF. An RCT was therefore designed to compare SPAIRE with the well-documented DA approach in HA for FNF, hypothesizing no differences between them. Data on bone loss around the femoral component and association between biomarkers indicating muscle damage and clinical outcomes have been published^14,15^. We now report 12-month clinical outcomes.

## Materials and Methods

**Fig. 1:**
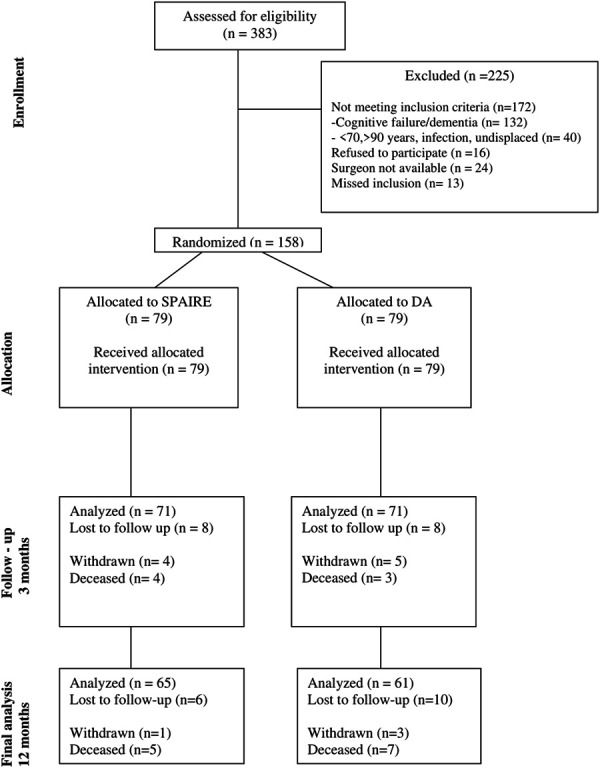
CONSORT diagram for the recruitment and flow of study participants. CONSORT = Consolidated Standards of Reporting Trials, DA = direct anterior, and SPAIRE = sparing piriformis and obturator internus, repair externus.

158 FNF patients were between January 2022 and July 2024 included in this blinded level 1 prospective RCT comparing the SPAIRE approach and DA approach in HA (Fig. 1). Patients were included at Sorlandet Hospital Kristiansand and Sorlandet Hospital Arendal in 2 parallel intervention groups (1:1 ratio) according to randomization and operated with cemented HA (Table I). Primary outcome measure was Harris Hip Score (HHS)^16^ at 3 and 12 months. Secondary outcome measures were hip disability and osteoarthritis outcome score (HOOS)^17^, EQ 5D^18^, pain and patient satisfaction measured with visual analog scale (VAS), and Timed Up & Go test (TUG test)^19^. Data regarding race and ethnicity were not collected as consideration of these variables was not part of the original study design.

**TABLE I:** Inclusion and Exclusion Criteria

Inclusion Criteria	Exclusion Criteria
Age 70-90	Dementia
Dislocated FNF	Pathologic fracture
Low-energy fracture	Retained hardware interfering with HA
Ambulatory prior to fracture	Sepsis or local infection

FNF = femoral neck fracture, and HA = hemiarthroplasty.

### Randomization

After informed consent, eligible patients were included by the on-call surgeon and randomized to one of the surgical approaches using a computer-generated, site-stratified randomization sequence prepared by a statistician. Allocation used sealed, lightproof envelopes with random block sizes^2-10^ containing even numbers within each block. All participants were analyzed according to their randomized allocation (Table II).

**TABLE II:** Patient Characteristics

	SPAIRE (n = 79)	DA (n = 79)
Age (SD)	80 (6)	79 (5)
Male (%)	26 (33)	38 (48)
Female (%)^*^	53 (67)	41 (52)
ASA ½ (%)	26 (33)	31 (39)
ASA ¾ (%)	53 (67)	48 (61)
BMI (SD)	24 (4)	25 (3)
HHS (SD)	89 (13)	90 (13)

ASA = American Society of Anesthesiologists Classification, BMI = body mass index, DA = direct anterior, HHS = Harris hip score preoperatively, and SPAIRE = sparing piriformis and obturator internus repair externus.

* Sex distribution showed a borderline imbalance between treatment arms (p = 0.051).

## Surgery and Implants

Patients were operated according to randomization within 48 hours by an orthopaedic surgeon well familiar with both the approaches. The C-STEM AMT (DePuy Synthes, Warsaw, IN) was used together with a 28 mm ARTICULEZE femoral head and the SELF-CENTERING Bi-polar Head (both from DePuy Synthes). Palacos R + G (Heraeus) cement was used. Patients in the DA group were operated in supine position without traction and the SPAIRE approach was performed in the lateral decubitus position.

Patients underwent surgery under spinal anesthesia, received 4 doses of cefazolin (2 g every 3 hours) and 1 g of tranexamic acid (unless contraindicated). Mobilization was attempted on the day of surgery, and physiotherapy started on day 1 postoperatively. Both treatment groups received identical postoperative care, including the same analgesic protocol. Preoperative HHS was recorded as baseline. Study personnel were blinded to outcome measures. HOOS, VAS pain, VAS satisfaction, and EuroQol 5-Dimension (EQ-5D) questionnaires were sent to patients before 3- and 12-month appointments. PROMs were collected by blinded physiotherapists, who also performed HHS and TUG tests.

## Statistics

This study compared the SPAIRE and DA approaches in patients with FNF undergoing HA, with HHS as the primary outcome. Sample size calculation was based on published studies on assumed SD of 13 to 15 using HHS and a minimal clinical important difference (MCID) of 7 to 10^20,21^. With an estimated SD of 13 and an MCID of 7 (5% significance), 73 patients were required per group for 90% power. Accounting for an estimated 20% 12-month mortality and 10% dropout, 190 patients were planned for inclusion. Owing to delayed recruitment at 1 hospital and fewer FNF cases, possibly related to the COVID-19 pandemic, statistical power was reduced to 80%. Before analyzing any outcome measures, and without access to unblinded data, the recruitment target was revised to 80%. The primary end point, target effect size, alpha level and statistical analysis plan were unchanged. The decision to reduce power from 90% to 80% was thoroughly discussed in the study group and was solely done due to recruitment challenges at one of the recruiting hospitals. Including a new hospital for recruitment was discussed, but not possible as no other Norwegian hospital operated FNFs with the 2 approaches. This reduced the necessary sample size from 190 to 141 (including estimated loss to follow-up). To reduce the risk of underpowering we included 158 patients. The groups were analyzed by intention to treat principles. Histograms, Q-Q plots, and the Shapiro-Wilk test were used to investigate for normality. Normally distributed variables were analyzed using Student *t* test and the Mann-Whitney *U*-test was used for non-normally distributed variables. A linear regression model was used on outcome measures collected only at follow-up (EQ-5D, HOOS). Our primary analyses used linear (parametric) models because these methods are widely used for EQ-5D and HOOS outcomes in orthopaedic and trauma research and because inference in linear models depends primarily on the distribution of residuals rather than the raw outcome distribution. Model assumptions were evaluated by inspection of residual distributions and residual plots, which did not indicate substantial deviations from normality or heteroscedasticity. To ensure that our conclusions were not dependent on parametric assumptions, we performed sensitivity analyses using both nonparametric group comparisons (Mann-Whitney *U* tests) and bootstrapped confidence intervals for the adjusted regression models (1,000 resamples, bias-corrected and accelerated method). Missing data were expected due to the inclusion criteria and patient characteristics (Table I). The Little Missing Completely at Random (MCAR) test was used to evaluate whether the missing data were random and unrelated to observed or nonobserved variables. For our main outcome, HHS, we found a Little’s MCAR: χ2 of p = 0.244. This indicated that missing HHS data were MCAR and HHS is reported without adjustments. Sex distribution showed a borderline imbalance between treatment arms (p = 0.051) and should be accounted for. This discrepancy likely reflects eligibility-related exclusions or between-site variations in patient demographics.

All analyses were reviewed by a blinded statistician at Oslo Centre of Biostatistics and Epidemiology, Norway. A p-value ≤ 0.05 was considered statistically significant. SPSS Statistics 21 for Windows (IBM Corp, Armonk, NY) was used for statistical analysis.

## Ethics, Registration, Funding, and Conflicts of Interest

The trial was approved by the Regional Ethics Committee, South-East Norway, on March 15, 2021(153,700), financed by the Norwegian Health Authority and registered at Clinical Trials.gov (NCT04900506). All patients were included after signed consent according to the principles of the Helsinki guidelines, and the study was reported according to the principles of the Consolidated Standards of Reporting Trials statement^22^.

## Results

The overall mean age was 79 (range 70-90), and 59% were women (Table II). 158 patients were enrolled, and 126 attended final 12-month follow-up (Fig. 1). The mean operating time was 63 minutes (41-109) in the DA group and 53 minutes (37-70) in the SPAIRE group (mean difference 9 minutes, 95% confidence interval [CI] 8-15; p < 0.01).

**Fig. 2:**
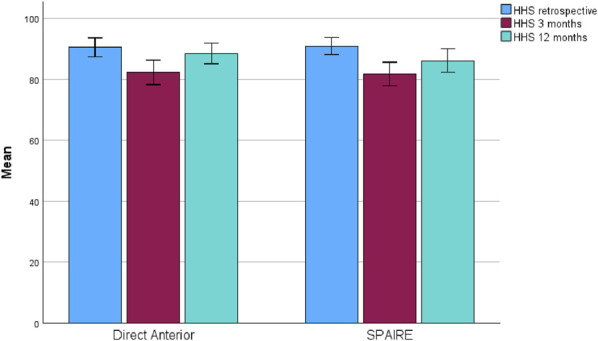
HHS boxplot at baseline (retrospective), 3- and 12-months follow-up. Error-bars represent 95% CI of mean. CI = confidence interval, HHS = Harris hip score.

The mean HHS was 82 (95% CI 78-86) in both groups at 3 months. HHS increased to 86 (95% CI 82-90) in the SPAIRE group and 88 (95% CI 85-92) in the DA group at 12 months (Table III and Fig. 2).

**TABLE III:** Summary of Clinical Outcomes (95% CI for Mean)

	No. of Patients SPAIRE DA	SPAIRE	DA	p
1. Postop D				
VAS pain	78 74	3.4 (2.9-3.9)	3.2 (2.5-3.6)	0.575
VAS satisfaction	76 74	2.2 (1.7-2.7)	2.2 (1.6-2.8)	0.996
3. Postop D				
TUG	75 74	41 (29-52)	36 (26-46)	0.395
VAS pain	75 74	2.3 (1.9-2.7)	2.2 (1.6-2.4)	0.819
VAS satisfaction	75 73	1.9 (1.4-2.3)	1.8 (1.4-2.3)	0.804
3 Mo				
HHS	71 71	82 (78-86)	82 (78-86)	0.422
TUG	75 74	12 (10-13)	12 (10-13)	0.356
EQ-5D	70 66	0.80 (0.75-0.86)	0.86 (0.81-0.89)	**0.021**
HOOS Total	69 66	76 (71-82)	78 (71-80)	0.601
VAS pain	69 69	1.6 (1.1-2.1)	1.1 (0.8-1.5)	0.116
VAS satisfaction	70 70	1.8 (1.1-2.2)	1.5 (1.0-1.9)	0.417
12 mo				
HHS	65 61	86 (82-90)	88 (85-92)	0.365
TUG	65 60	12 (10-14)	11 (9-12)	0.208
HOOS total	62 55	80 (75-86)	80 (75-84)	0.449
EQ-5D	62 57	0.82 (0.76-0.88)	0.83 (0.79-0.87)	0.670
VAS pain	64 58	1.0 (0.5-1.4)	1.1 (0.7-1.6)	0.729
VAS satisfaction	63 59	1.2 (0.8-1.8)	1.3 (0.8-1.9)	0.845

CI = confidence interval, DA = direct anterior, EQ-5D = EuroQol 5-dimension, HHS = Harris hip score, HOOS = hip disability and osteoarthritis outcome score, SPAIRE = sparing piriformis and obturator internus repair externus, TUG = timed up & go, and VAS = visual analog scale. Bold values indicate statistical significance, p < 0.05.

EQ-5D was only measured at 3- and 12-month follow -up. The SPAIRE group had a lower mean score (0.80 ± 0.22) than the DA group (0.86 ± 0.14), (95% CI 0.01-0.14, p = 0.024) at 3 months. TUG test improved from 41 seconds (95% CI 29-52) on day 3 in the SPAIRE group to 12 seconds (95% CI 10-13) at 3 months. In the DA group TUG-test improved from 36 seconds (95% CI 26-46) on day 3 to 12 seconds (95% CI 10-13) at 3 months (Table III). TUG-test at 12 months were 12 seconds (95% CI 10-14) and 11 seconds (95% CI 9-11), respectively (Table III). No significant differences were observed between the groups regarding VAS pain, VAS patient satisfaction, and HOOS scores (Table III).

## Adverse Events

During the 12-month follow-up period, a total of 4 infections (2 in each group), 2 dislocations (1 in each group), and 1 low-energy periprosthetic fracture (DA group) were recorded. 5 patients were registered with damage to the lateral cutaneous femoral nerve at 3- month follow-up, 3 patients had persistent hypoesthesia at 12 months. One patient was reoperated due to an oversized bipolar head, and 1 patient was admitted 4 months postoperatively with a sacral pressure ulcer requiring surgical debridement.

## Discussion

This RCT comparing DA and SPAIRE approaches in displaced FNF patients treated with cemented HA showed equal HHS at 12 months. EQ-5D favored DA at 3 months, but the 0.08 difference was below the MCID of 0.2 found by Langenberger et al.^23^ indicating no clinically meaningful benefit. No differences were observed in HHS, HOOS, VAS, or TUG at 3 or 12 months nor in major complications. However, the study was not powered to detect differences in complication rates, and we cannot exclude that potential differences in PROMS may have been present between discharge and the 3-month follow-up. The primary objective was comparison of PROMs between the approaches.

PA in HA has an increased risk of dislocation and Norwegian guidelines advocate against the PA in HA for FNF^24^. Studies have reported decreased dislocation rates by sparing the piriformis tendon from detachment^25,26^. A few reports regarding the SPAIRE approach have been published indicating that stability improves when the obturator internus tendon is spared in addition to the piriformis tendon^10,11^. Since the introduction in Norway in 2018 the SPAIRE approach in HA has become increasingly popular^1^. 18% of HA’s in 2024 were done using the SPAIRE approach, although the evidence of reduced dislocation risk is scarce. We registered 1 dislocation in each group (1.3%). Patients with existing cognitive impairment before fracture were excluded from participation. This excludes a high-risk population and probably underestimates the dislocation rate. PROMs reflecting patient satisfaction and quality of life are essential when comparing treatments, and our results do not support superiority of either surgical approach. Khan et al. reported improved early postoperative outcomes with the DA approach compared with DL, anterolateral, and PA in HA for FNF patients^9^, whereas Gusho et al. found no clinical differences among these approaches^27^. Regarding SPAIRE, Charity et al. reported increased odds of returning to pre-injury mobility levels in FNF patients treated with this approach^13^. Ball et al. reported less pain in the early postoperative period in favor of SPAIRE compared with DL approach, but no difference at 3-month follow-up. They also reported complication rates comparable with the DL approach^11^.

The strength of this study is that it is the first RCT comparing a well-documented DA approach and a novel SPAIRE approach in HA for FNF. The treatment groups were well balanced. The study was performed at 2 hospitals and patients were examined by blinded physiotherapists. Missing data was within pre-estimations in an old and frail cohort.

Limitations include the reduction of power, accounted for in this article, from 90% to 80%. Another limitation is the exclusion of patients with preexisting dementia. This patient group has a higher risk of adverse events, and this should be accounted for when interpreting the results. The absence of descriptive data regarding race and ethnicity is a limitation that may affect the study's generalizability. There was a borderline imbalance between treatment arms (p = 0.051) regarding sex distribution and this should be accounted for. This discrepancy likely reflects eligibility-related exclusions or between-site variations in patient demographics.

## Conclusion

This RCT compared the SPAIRE and DA approaches and found no significant differences in clinical outcomes. The SPAIRE approach may be an alternative for PA surgeons performing HA on FNF patients, although larger studies are needed to confirm its clinical implications.

## Data Availability

A data sharing statement is provided with the online version of the article.

## References

[1] 2023 Norwegian Arthroplasty Register. Annual Report; 2023. https://www.helse-bergen.no/48d1eb/contentassets/9f19d57711ee4e60815d6b89e8e8472b/report2024.pdf.

[2] Svenoy S Westberg M Figved W Valland H Brun OC Wangen H Madsen JE Frihagen F. Posterior versus lateral approach for hemiarthroplasty after femoral neck fracture: early complications in a prospective cohort of 583 patients. Injury. 2017;48(7):1565-9. 28465004 10.1016/j.injury.2017.03.024

[3] Leonardsson O Rolfson O Rogmark C. The surgical approach for hemiarthroplasty does not influence patient-reported outcome: a national survey of 2118 patients with one-year follow-up. Bone Joint J. 2016;98-b(4):542-7. 27037438 10.1302/0301-620X.98B4.36626

[4] Amlie E Havelin LI Furnes O Baste V Nordsletten L Hovik O Dimmen S. Worse patient-reported outcome after lateral approach than after anterior and posterolateral approach in primary hip arthroplasty. Acta Orthopaedica. 2014;85(5):463-9. 24954494 10.3109/17453674.2014.934183 PMC4164862

[5] Mjaaland KE Kivle K Svenningsen S Nordsletten L. Do postoperative results differ in a randomized trial between a direct anterior and a direct lateral approach in THA? Clin Orthop Relat Res. 2019;477(1):145-55. 30179928 10.1097/CORR.0000000000000439 PMC6345297

[6] Masonis JL Bourne RB. Surgical approach, abductor function, and total hip arthroplasty dislocation. Clin Orthop Relat Res. 2002;405(405):46-53. 10.1097/00003086-200212000-00006 12461355

[7] Ugland TO Haugeberg G Svenningsen S Ugland SH Berg ØH Pripp AH Nordsletten L. High risk of positive trendelenburg test after using the direct lateral approach to the hip compared with the anterolateral approach: a single-centre, randomized trial in patients with femoral neck fracture. Bone Joint J. 2019;101-b(7):793-9. 31256660 10.1302/0301-620X.101B7.BJJ-2019-0035.R1 PMC6617057

[8] Kunkel ST Sabatino MJ Kang R Jevsevar DS Moschetti WE. A systematic review and meta-analysis of the direct anterior approach for hemiarthroplasty for femoral neck fracture. Eur J Orthop Surg Traumatol. 2018;28(2):217-32. 28852880 10.1007/s00590-017-2033-6

[9] Khan IA Magnuson JA Arshi A Krueger CA Freedman KB Fillingham YA. Direct anterior approach in hip hemiarthroplasty for femoral neck fractures: do short-term outcomes differ with approach?: a systematic review and meta-analysis. JBJS Rev. 2022;10(9):e21.00202. 10.2106/JBJS.RVW.21.00202 36053029

[10] Nakamura T Yamakawa T Hori J Goto H Nakagawa A Takatsu T Naoki Osamura Saito A Keisuke Hagio Mouri K. Conjoined tendon preserving posterior approach in hemiarthroplasty for femoral neck fractures: a prospective multicenter clinical study of 322 patients. J Orthop Surg (Hong Kong). 2021;29(3):23094990211063963. 34920684 10.1177/23094990211063963

[11] Ball S Aylward A Cockcroft E Corr A Gordon E Kerridge A McAndrew A Morgan-Trimmer S Powell R Price A Rhodes S Timperley AJ van Horik J Wickins R Charity J. Clinical effectiveness of a modified muscle sparing posterior technique compared with a standard lateral approach in hip hemiarthroplasty for displaced intracapsular fractures (HemiSPAIRE): a multicenter, parallel-group, randomized controlled trial. BMJ Surg Interv Health Tech. 2024;6(1):e000251. 10.1136/bmjsit-2023-000251 PMC11184196 38895600

[12] Han SK Kim YS Kang SH. Treatment of femoral neck fractures with bipolar hemiarthroplasty using a modified minimally invasive posterior approach in patients with neurological disorders. Orthopedics. 2012;35(5):e635-40. 22588403 10.3928/01477447-20120426-15

[13] Charity J Ball S Timperley AJ. The use of a modified posterior approach (SPAIRE) may be associated with an increase in return to pre-injury level of mobility compared to a standard lateral approach in hemiarthroplasty for displaced intracapsular hip fractures: a single-centre study of the first 285 cases over a period of 3.5 years. Eur J Trauma Emerg Surg. 2023;49(1):155-63. 35879617 10.1007/s00068-022-02047-1 PMC9925473

[14] Ugland SH Fagerberg OT Mjaaland KE Ugland TO Haugeberg G Pripp AH Nordsletten L. No benefit in biomarkers assessing muscle damage for minimally invasive anterior over SPAIRE approach in hemiarthroplasty: a subgroup analysis with 100 patients from a randomized controlled trial. Bone Joint Open. 2025;6(10):2032-8. 41067724 10.1302/2633-1462.610.BJO-2025-0027.R1 PMC12516294

[15] Ugland SH Ugland TO Mjaaland KE Fagerberg OT Haugeberg G Pripp AH Nordsletten L. SPAIRE approach shows equivalent changes in bone mineral density as a conventional approach in femoral neck fracture patients: a sub-group analysis of 49 patients from a randomized controlled trial. Bone Joint Open. 2025;6(4):398-405. 40185485 10.1302/2633-1462.64.BJO-2024-0171.R1 PMC11970974

[16] Harris WH. Traumatic arthritis of the hip after dislocation and acetabular fractures: treatment by mold arthroplasty. An end-result study using a new method of result evaluation. J Bone Joint Surg Am. 1969;51(4):737-55. 5783851

[17] Nilsdotter AK Lohmander LS Klässbo M Roos EM. Hip disability and osteoarthritis outcome score (HOOS)--validity and responsiveness in total hip replacement. BMC Musculoskelet Disord. 2003;4(1):10. 12777182 10.1186/1471-2474-4-10 PMC161815

[18] Kasuya A. EuroQol--a new facility for the measurement of health-related quality of life. Health Policy. 1990;16(3):199-208. 10109801 10.1016/0168-8510(90)90421-9

[19] Podsiadlo D Richardson S. The timed “Up & Go”: a test of basic functional mobility for frail elderly persons. J Am Geriatr Soc. 1991;39(2):142-8. 1991946 10.1111/j.1532-5415.1991.tb01616.x

[20] Frihagen F Nordsletten L Madsen JE. Hemiarthroplasty or internal fixation for intracapsular displaced femoral neck fractures: randomised controlled trial. BMJ. 2007;335(7632):1251-4. 18056740 10.1136/bmj.39399.456551.25 PMC2137068

[21] Figved W Opland V Frihagen F Jervidalo T Madsen JE Nordsletten L. Cemented versus uncemented hemiarthroplasty for displaced femoral neck fractures. Clin Orthop Relat Res. 2009;467(9):2426-35. 19130162 10.1007/s11999-008-0672-y PMC2866935

[22] Schulz KF Altman DG Moher D. CONSORT 2010 statement: updated guidelines for reporting parallel group randomised trials. BMJ. 2010;340:c332. 20332509 10.1136/bmj.c332 PMC2844940

[23] Langenberger B Steinbeck V Busse R. Who benefits from hip arthroplasty or knee arthroplasty? Preoperative patient-reported outcome thresholds predict meaningful improvement. Clin Orthop Relat Res. 2024;482(5):867-81. 38393816 10.1097/CORR.0000000000002994 PMC11008644

[24] Tellefsen R Kristensen TB Dybvik EH Gjertsen JE Nordsletten L Ugland T Visnes H Solberg LB. Surgical approaches in hemiarthroplasty for hip fracture: results of 48,222 hemiarthroplasties in the Norwegian hip fracture register. Bone Joint Open. 2025;6(10):1311-20. 41120102 10.1302/2633-1462.610.BJO-2025-0099.R1 PMC12539978

[25] Viberg B Kristensen EQ Gaarsdal T Petersen CD Jensen TG Overgaard S . A piriformis-preserving posterior approach reduces dislocation rate of the hemiarthroplasty in patients with femoral neck fracture. Injury. 2023. 10.1016/j.injury.2023.04.040 37100693

[26] Apinyankul R Satravaha Y Mokmongkolkul K Phruetthiphat OA. Comparison of dislocation and outcome between piriformis-sparing and conventional posterior approach after bipolar hemiarthroplasty for femoral neck fracture in patients over 60 years. J Arthroplasty. 2023;38(4):732-6. 36273711 10.1016/j.arth.2022.10.025

[27] Gusho C Hoskins W Ghanem E. A comparison of surgical approaches for hip hemiarthroplasty performed for the treatment of femoral neck fracture: a systematic review and network meta-analysis of randomized controlled trials. JBJS Rev. 2024;12(6). 10.2106/JBJS.RVW.24.00067 38889234

